# Genetically Modified T-cell Therapy for the Treatment of Osteosarcoma: An Update

**DOI:** 10.4172/2155-9899.1000417

**Published:** 2016-04-29

**Authors:** Christopher DeRenzo, Stephen Gottschalk

**Affiliations:** 1 Center for Cell and Gene Therapy, Texas Children’s Hospital, Houston Methodist Hospital, Baylor College of Medicine, Houston, Texas 77030, USA; 2 Texas Children’s Cancer Center, Texas Children’s Hospital, Baylor College of Medicine, Houston, Texas 77030, USA; 3 Department of Pediatrics Baylor College of Medicine, Houston, Texas 77030, USA; 4 Department of Pathology and Immunology, Baylor College of Medicine, Houston, Texas 77030, USA

**Keywords:** Pediatric cancer, Osteosarcoma, Cancer immunotherapy, T-cell therapy, Gene therapy, Chimeric antigen receptors, Tumor antigens

## Introduction

**Figure 1:**
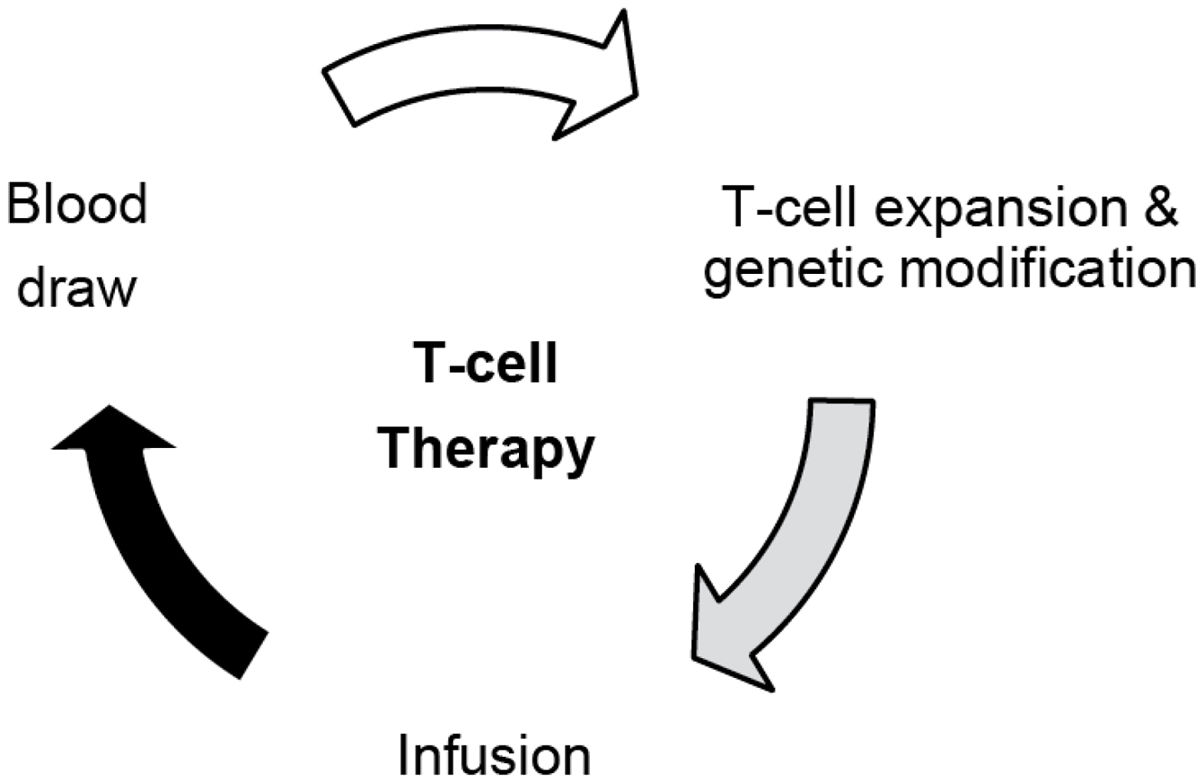
Cell therapy with genetically modified T cells. Blood is drawn from patients, T cells are expanded and genetically modified in the laboratory before they are reinfused into patients.

While patients with localized osteosarcoma have excellent survival rates, the prognosis for those with advanced disease is significantly worse despite aggressive multimodality therapies [1]. To overcome these limitations novel treatments are needed, especially for children and young adults with metastatic and/or recurrent osteosarcoma, whose poor rates of survival have remained relatively unchanged for decades. Immunotherapy with genetically modified T cells (Figure 1) offers a promising alternative to conventional cytotoxic chemotherapies because T-cell killing does not rely on mechanisms employed by these cytotoxic agents [2,3].

In this communication, we briefly summarize our recent article detailing genetically modified T-cell therapy for osteosarcoma [4], as well as describe our experience using HER2-CAR T cells in a phase I clinical trial for patients with HER2-positive osteosarcoma [5]. Furthermore, we highlight additional immunomodulatory maneuvers that may enhance the efficacy of genetically modified T-cell therapy for the treatment of patients with osteosarcoma.

## Genetically Modified T cells for Osteosarcoma: A Brief Review

Successful gene transfer strategies include the forced expression of antigen-specific α/β T-cell receptors (TCRs) or chimeric antigen receptors (CARs) [6]. Conventional TCRs are composed of α and β chains, and recognize peptide fragments presented on major histocompatibility complex (MHC) molecules. Compared to genetically modifying T cells with CARs, generating tumor-specific T cells by forced expression of TCRs is cumbersome. Despite the inherent difficulty of synthesizing large numbers of α/β TCR-modified T cells, clinical studies using this approach demonstrate the potency of adoptively transferred α/β TCR-modified T cells for multiple cancer types, including NY-ESO-1-specific T cells for patients with synovial sarcoma [7–11]. Therefore active exploration of α/β TCR-modified T-cell therapy is warranted for patients with osteosarcoma.

**Figure 2:**
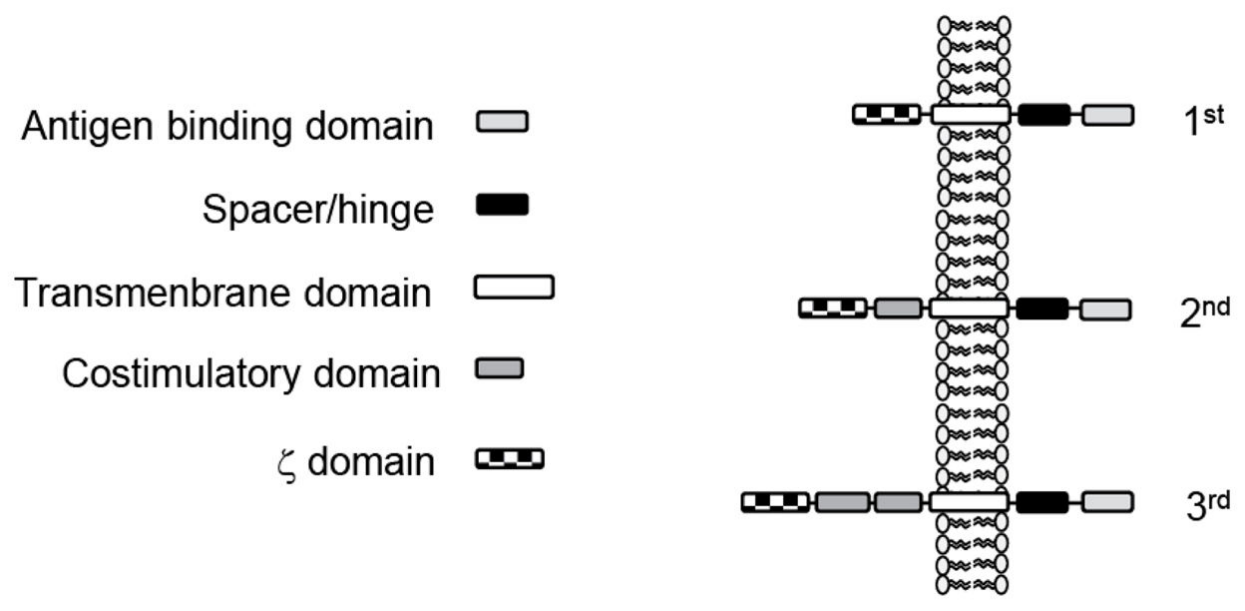
Basic design of chimeric antigen receptors. Chimeric antigen receptors (CARs) consist of an antigen binding domain, a spacer/hinge region, a transmembrane domain, and an endodomain that consists of domains derived from costimulatory molecules and CD3-z. Depending on the number of costimulatory endodomains, CARs are designated as 1^st^ generation (no costimulatory domain), 2^nd^ generation (one costimulatory domain), 3^rd^ generation (two costimulatory domain).

CAR T cells have several advantages over α/β TCR T cells because CAR T cells recognize and kill tumor cells in a MHC unrestricted fashion. Therefore, target recognition by CAR T cells is unaffected by some of the major mechanisms by which tumors avoid MHC-restricted T-cell (α/β TCR) recognition, such as downregulation of HLA class I molecules and defective antigen processing. CARs consist of an extracellular domain (ectodomain) that confers antigen specificity, a hinge region, transmembrane domain, and an intracellular domain (endodomain) [12]. The most commonly used method to generate extracellular CAR domains is by joining the heavy and light chain variable regions derived from monoclonal antibodies. Because CARs are derived from monoclonal antibodies, CAR T cells combine the antigen-binding property of monoclonal antibodies with the lytic capacity and self-renewal of T cells. CAR endodomains are typically derived from the T-cell receptor CD3-ζ chain, which can be combined with costimulatory molecules such as CD28 or 41BB. CAR nomenclature is based on the number of costimulatory domains contained within the intracellular CAR construct. CARs containing zero costimulatory domains are dubbed first generation, one costimulatory domain denotes second generation, and two costimulatory domains denotes third generation CARs (Figure 2).

Of the multiple tumor associated antigens expressed by osteosarcoma cells, human epidermal growth factor receptor (HER)2, disialoganglioside (GD)2, interleukin (IL)11Rα, fibroblast activation protein, and B7-H3 [13–17] are expressed on the tumor cell surface, making them viable CAR T-cell targets. We have focused on targeting HER2 with CAR T cells. Although osteosarcoma tumors are often HER2-positive, the HER2 gene is not amplified in this disease [18]. Thus, osteosarcoma is part of a group of tumors that express HER2 at levels too low for HER2 monoclonal antibodies to be effective [19]. We and others have shown that malignancies that express HER2 at low levels can be targeted with T cells that express HER2-CARs [13], and recently implemented a phase I clinical study using HER2-CAR T cells for the immunotherapy of HER2-positive sarcomas [5], with most patients enrolled having a diagnosis of osteosarcoma.

## HER2-CAR T cells for Patients with Osteosarcoma

In a phase I clinical trial we evaluated the feasibility and safety of administering escalating doses of 2^nd^ generation HER2-CAR (HER2.CD28.ζ-CAR) T cells for patients with recurrent/refractory HER2-positive sarcomas [5]. Sixteen of 19 patients who received HER2-CAR T cells were diagnosed with osteosarcoma. We began our study with an ultra-low dose of HER2-CAR T cells (1 × 10^4^/m^2^) and escalated over eight dose levels to a maximum dose of 1 × 10^8^/m^2^ T cells. HER2-CAR T cells were successfully generated for all patients. In regards to safety, none of the patients had adverse events related to the T-cell infusion, except for one patient on the highest dose level, who developed fever within 12 hours after T-cell infusion, which resolved with ibuprofen. Additionally, all patients enrolled had pre-infusion echocardiograms demonstrating normal left ventricular ejection fraction (LVEF), and the LVEF remained normal in all patients six weeks after T-cell infusion. In combination, these results demonstrate the feasibility of generating HER2-CAR T cells from the peripheral blood of patients previously treated with multi-agent cytotoxic chemotherapies, and illustrate the safety of HER2-CAR T cells administered to patients with HER2-positive osteosarcoma at doses up to 1 × 10^8^/m^2^.

Because efficacy of CAR T cells depends upon persistence of the infused T-cell product [20], we assessed persistence of HER2-CAR T cells by quantitative polymerase chain reaction analysis of peripheral blood mononuclear cells. In patients who received T-cell doses from 1 × 10^5^/m^2^ and higher, we detected HER2-CAR T cells in the peripheral blood of 14 of 16 patients, and the copy number correlated with the infused T-cell dose. Despite our ability to detect HER2-CAR T cells, 3 hours after T-cell infusion there was a rapid decline in the frequency of HER2-CAR T cells in the peripheral blood of all patients, demonstrating a lack of T-cell expansion. Despite the lack of expansion, low-levels of HER2-CAR T cells were detected 6 weeks after infusion in 7 of 9 evaluable patients who received greater than 1 × 10^6^/m^2^ T cells. Furthermore, at 3 months we detected HER2-CAR T cells in 4 of 13 evaluable patients. Thus, although we found no evidence for HER2-CAR T cell expansion after infusion, HER2-CAR T cells persisted long term in some patients.

Clinical response to T-cell infusion was evaluated by comparing disease identified by imaging obtained before HER2-CAR T cell infusion to images obtained 6 weeks after infusion. Of 17 evaluable patients, 4 had stable disease for 12 weeks to 14 months. Three patients with stable disease received no additional therapy and had their residual tumor removed. A sample from one of these patients showed >90% necrosis, demonstrating antitumor activity of infused HER2-CAR T cells. All three patients remain in remission with no further treatment until now with a follow up of >3 years. Although HER2 expression was measured and objectively graded for all patients on this study, we were unable to establish a relationship between tumor HER2 expression and clinical response because the sample size was insufficient for such an analysis. In future clinical trials we plan to evaluate whether HER2-CAR T cells have greater efficacy for patients with high HER2-expressing osteosarcoma compared to those expressing relatively low HER2 levels. Data from this study show that a safe dose of HER2-CAR T cells can be established for patients with HER2-positive osteosarcoma, and these cells can persist at low levels for more than 6 weeks in a dose-dependent manner. Despite these encouraging findings, clinical benefit of HER2-CAR T cells was limited, indicating that further manipulation of the immune system will be essential to enhance outcomes for patients with osteosarcoma.

## Immune Modulation to Enhance T-cell Therapy for Osteosarcoma

T-cell expansion and long-term persistence of adoptively transferred T cells has been observed in patients receiving lymphodepleting chemotherapy [21]. Due to serious safety concerns, which arose after a single patient developed fatal acute respiratory failure following the administration of 1 × 10^10^ third generation HER2-CAR T cells, derived from the monoclonal antibody trastuzumab, in combination with IL-2 and lymphodepleting chemotherapy [22], we conducted our clinical study with HER2-CAR T cells without lymphodepletion. Since we have now established a safe dose (1 × 10^8^/m^2^) of HER2-CAR T cells, we are currently evaluating the safety and clinical benefits of lymphodepleting patients with fludarabine +/− cyclophosphamide prior to the infusion of 1 × 10^8^/m^2^ HER2-CAR T cells.

An alternative, or perhaps complimentary strategy, to enhance the efficacy of CAR T-cells for osteosarcoma is to combine CAR T cells with one or more checkpoint antibodies now becoming available for cancer immunotherapy. Intriguingly, Lussier and colleagues demonstrated in human osteosarcoma samples that metastatic but not primary osteosarcoma tumors express the T-cell inhibitory ligand program death ligand 1 (PD-L1) [23]. They also provide evidence that cytotoxic T cells infiltrating human metastatic osteosarcomas upregulate programmed death receptor 1 (PD-1), implicating this T-cell inhibitory pathway as a contributing factor to osteosarcoma induced suppression of cytotoxic T cells. Furthermore, blocking the PD-1/PD-L1 pathway using monoclonal antibody resulted in decreased tumor burden and increased survival in a murine osteosarcoma model system [23]. In a separate article, Lussier and colleagues demonstrate that T cells infiltrating osteosarcoma tumors upregulate CTLA-4, another receptor involved in decreasing activation of cytotoxic T-cells [24]. Based on these findings osteosarcoma bearing mice were treated with antibodies against both PD-L1 and CTLA-4. This combinational therapy significantly enhanced anti-osteosarcoma activity compared to mice that received either antibody alone. Importantly, a phase I clinical trial is currently underway to test the safety and efficacy of PD-1 antibody alone, or in combination with CTLA-4 antibody for the treatment of children, adolescents and young adults with osteosarcoma and other solid tumors (NCT02304458). In a separate phase I clinical trial, GD-2 CAR T cells given in combination with PD-1 antibody is being evaluated for patients with relapsed/refractory neuroblastoma (NCT01822652). Targeting PD-L1 directly with PD-L1-speciifc CAR T cells is not an option since PD-L1 is expressed on a broad array of normal cells, however investigators have shown that it is possible to express chimeric PD-1 receptors on the cell surface of T cells that consist of the PD-1 ecto- and transmembrane domains, and the CD28 costimulatory endodomain to convert the ‘negative’ PD-L1 into a ‘positive’ costimulatory signal [25]. Given these findings, clinical trials using PD-1 and/or CTLA-4 antibody in combination with genetically modified T cells for the immunotherapy of osteosarcoma are likely to be developed in the near-term future.

Another method to disrupt the T-cell inhibitory PD-1/PD-L1 axis is by genetically modifying T cells to silence PD-1 expression or disrupt the PD-1 gene locus. Su and colleagues demonstrate that PD-1 can successfully be downregulated using a state of the art CRISPR/Cas9 immune editing system [26]. Reducing PD-1 did not affect the viability or persistence of primary human T cells cultured *in vitro*. Furthermore, immune responses of T cells genetically modified to downregulate PD-1 were enhanced, as evidenced by increased IFN-γ secretion upon recognition of target antigen, and enhanced killing of PD-L1 positive melanoma cells. Thus disrupting the PD-1 gene locus in CAR T cells has the potential to render T-cells resistant to osteosarcoma induced immunosuppression, and enhance anti-osteosarcoma activity.

## Beyond Genetic Modification That Renders T cells Tumor Antigen Specific

As already discussed in the last section, a ‘2^nd^ genetic modification’ of CAR T cells holds the promise to further enhance antitumor activity. Conceptually these can be divided into strategies to i) overcome immune escape by targeting multiple tumor antigens, ii) enhance T-cell expansion and persistence by transgenic expression of cytokines, cytokine receptors, chimeric cytokine receptors or silencing negative regulators, iii) increase T-cell trafficking to tumors, iv) render T cells resistant to the immunosuppressive tumor microenvironment, and v) to increase safety. With few exceptions [27,28], these strategies have been mainly evaluated in preclinical models with encouraging results. Several detailed reviews have been recently published on this topic [29–34], and we refer the interested reader to these publications.

## Conclusion

Genetically modified T cells have shown potent antitumor activity in multiple preclinical osteosarcoma models, and initial safety data in the first clinical experience using HER2-CAR T cells for patients with HER2-positive osteosarcoma is encouraging. However, several challenges remain including limited *in vivo* CAR T-cell expansion and persistence, and overcoming the inhibitory tumor microenvironment. We and others believe that CAR T-cell therapy for the treatment of patients with osteosarcoma remains promising. However, combinatorial immune modulating therapies and/or additional genetic modification strategies will be necessary before CAR T cells will have a major impact in the clinical management of this devastating solid tumor.
